# Arbidol is associated with increased in-hospital mortality among 109 patients with severe COVID-19: A multicenter, retrospective study

**DOI:** 10.7189/jogh.11.05017

**Published:** 2021-07-17

**Authors:** Xianlong Zhou, Haifeng Hou, Luyu Yang, Guoyong Ding, Tao Wei, Cancan Li, Yuanyuan Heng, Ruining Liu, Min Ma, Zhuanzhuan Hu, Lei Huang, Xizhu Xu, Quan Hu, Yan Zhao, Weijia Xing, Zhigang Zhao

**Affiliations:** 1 Emergency Center, Zhongnan Hospital of Wuhan University, Wuhan, Hubei, China; 2 School of Public Health, Shandong First Medical University & Shandong Academy of Medical Sciences, Tai’an, Shandong, China; 3 School of Public Health and Management, Binzhou Medical University, Yantai, China; 4 Intensive Care Unit, Wuhan Third Hospital, Wuhan, Hubei, China; 5 Intensive Care Unit, Huazhong University of Science and Technology Union Jiangbei Hospital, Wuhan, Hubei, China; 6 Intensive Care Unit, The First People’s Hospital of Jiangxia District, Wuhan, Hubei, China; 7 Hubei Clinical Research Center for Emergency and Resuscitation, Zhongnan Hospital of Wuhan University, Wuhan, Hubei, China

## Abstract

**Background:**

The antiviral therapy has been considered as an ordinary intervention for COVID-19 patients. However, the effectiveness of antiviral therapy is uncertain. This study was designed to determine the association between the antiviral therapy and in-hospital mortality among severe COVID-19 patients.

**Methods:**

This study enrolled severe COVID-19 patients admitted to four designated hospitals in Wuhan, China. The use of antiviral treatments, demographics, laboratory variables, co-morbidities, complications, and other treatments were compared between survival and fatal cases. The association between antiviral agents and in-hospital mortality were analyzed.

**Results:**

In total, 109 severe COVID-19 patients (mean age 65.43) were enrolled for analysis, among which, 61 (56.0%) patients were discharged alive, and 48 (44.0%) died during hospitalization. We found no association between lopinavir/ritonavir (LPV/r) treatment and the in-hospital mortality (odds ratio (OR) = 0.195, 95% confidence interval (CI) = 0.023-1.679). Besides, ribavirin (OR = 0.738, 95% CI = 0.344-1.582), oseltamivir (OR = 0.765, 95% CI = 0.349-1.636), and interferon-alpha (IFN-α) (OR = 0.371, 95% CI = 0.112-1.236) were not associated with the in-hospital mortality. However, arbidol monotherapy (OR = 5.027, 95% CI = 1.795-14.074) or the combination of arbidol and oseltamivir (OR = 5.900, 95% CI = 1.190-29.247) was associated with an increased in-hospital mortality. In addition, the multiple logistic regression identified a significant association between the use of arbidol and the in-hospital mortality (adjusted OR = 4.195, 95% CI = 1.221-14.408).

**Conclusions:**

Our findings indicated that LPV/r, IFN-α, ribavirin, or oseltamivir have no beneficial effects on the prognosis of severe COVID-19 patients, whereas the use of arbidol is associated with increased in-hospital mortality.

The outbreak of coronavirus disease 2019 (COVID-19) caused by severe acute respiratory syndrome coronavirus 2 (SARS-CoV-2) has become an emergent global pandemic [1]. The clinical spectrum of COVID-19 can range from asymptomatic infection to multiple organ dysfunction requiring intensive care unit (ICU) admission [2,3]. Since no specific drug is available, current treatments for COVID-19 are mainly symptomatic and supportive [4]. In clinical practice, antiviral drugs such as ribavirin, lopinavir/ritonavir (LPV/r), oseltamivir, favipiravir, arbidol, and interferon (IFN) have been used for patients with coronavirus infections [5-8]. However, the effects of these antiviral drugs on the prognosis of severe COVID-19 patients have not been clearly clarified to date [9]. In the present study, we retrospectively analyzed 109 severe COVID-19 patients who were admitted to four designated hospitals in Wuhan City. We aim to determine whether the use of antiviral drugs reduces the in-hospital mortality in severe COVID-19 patients.

## METHODS

### Study setting

This retrospective, observational study was performed in 4 hospitals in Wuhan, China: Zhongnan Hospital of Wuhan University, Wuhan Third Hospital, Union Jiangbei Hospital and the First People's Hospital of Jiangxia District. This study was approved by the Medical Ethics Committee of these four hospitals.

### Study population

Severe COVID-19 patients from these hospitals between Jan 8 and Mar 9, 2020 were enrolled for analysis. A severe COVID-19 patient was defined as an oxygen saturation on room air at rest ≤93% or the partial pressure of oxygen in arterial blood/fraction of inspired oxygen ≤300 mm Hg (1 mm Hg = 0.133kPa) according to the guidelines of the National Health Commission (NHC) of China [10]. Inclusion criteria: male and nonpregnant female patients aged 18 years old or older were eligible if they were severe, had a laboratory confirmed infection of SARS-CoV-2, had radiological evidence of pneumonia and received antiviral drugs within 24 hours after hospital admission. Exclusion criteria: pregnant patients, patients aged <18 years old, or patients had no definite outcome at the time of enrollment.

### Outcomes

In-hospital mortality or survival at discharge was defined as the primary outcome of the study. The secondary outcomes included the intensive care unit (ICU) mortality and the development of complications.

### Data collection

The demographic data, signs and symptoms, co-morbidities, laboratory and radiological findings, use of antiviral drugs and other treatments, complications and outcome of all patients were collected from the electric medical records. Data were checked by two independent physicians, and a third expert made a final decision when disagreements occurred.

### Definition

Acute respiratory distress syndrome (ARDS) was determined according to the Berlin Definition [11]. Sepsis and septic shock were diagnosed according to the 2016 Third International Consensus Definition for Sepsis and Septic Shock [12]. Acute kidney injury (AKI) was diagnosed according to the KDIGO clinical practice guidelines [13], and acute cardiac injury was diagnosed by increased serum cardiac biomarkers. Disseminated intravascular coagulation (DIC) was defined according to the guidelines of the Scientific Subcommittee on Disseminated Intravascular Coagulation of the International Society on Thrombosis and Haemostasis [14].

### Laboratory testing for SARS-CoV-2

The throat swabs were collected for real-time reverse-transcription–polymerase-chain-reaction (RT-PCR) of the severe acute respiratory syndrome coronavirus 2 (SARS-CoV-2) using a commercial nucleic acid detection kit according to the manufacturer’s instructions (DAAN Gene Co., Ltd of Sun Yat-Sen University, Guangzhou, China) as previously described [15].

### Statistical analysis

We used mean and standard deviation (SD) for description of normally distributed continuous variables, and median and interquartile range (IQR) for continuous variables of unnormal distribution. We used number (N) and percentage (%) to describe categorical variables. We implemented χ^2^ (χ^2^) test to compared the in-hospital mortality, followed by evaluation of odds ratio (OR) and 95% confidence interval (CI), and to compare the frequency of the onsets of complications between groups. Meanwhile, we use non-parametric test (eg, Kruskal-Wallis test) to compare variables of unnormal distribution. We performed a multiple logistic regression analysis to identify the factors or antiviral agents that affect the outcomes of COVID-19 patients. All the analyses were performed with Prism (version 8.0, GraphPad Software, San Diego CA, USA) and R software (version 3.6.3, R Foundation for Statistical Computing, Vienna, Austria).

## RESULTS

### Demographic and clinical features

This study enrolled 109 severe, laboratory confirmed COVID-19 patients (mean age 65.43), who received antiviral therapy within 24 hours after hospital admission. Among of them, 106 (97.2%) patients have been admitted to ICU. With regard to primary outcome, 61 (56.0%) patients were discharged alive (survival group), while 48 (44.0%) patients died during hospitalization (non-survival group).

As shown in **Table 1**, no differences were observed in age, sex, or body mass index (BMI) between the survival and the non-survival group. The most common comorbidities of the patients were recorded including hypertension (46, 42.2%), diabetes (20, 18.3%), coronary heart disease (16, 14.7%), renal insufficiency (8, 7.3%), chronic lung disease (8, 7.3%), cerebrovascular disease (5, 4.6%), and malignant tumor (5, 4.6%). The non-survival group had a higher incidence of renal insufficiency than that in the survival group (14.6% vs 1.6%, *P* = 0.010). In addition, the incidence of fever (91.7% vs 75.4%, *P* = 0.026) and the median duration of fever (7 vs 5, *P* = 0.040) in the Non-survival group were significantly increased as compared to the survival group.

**Table 1:** Demographic and clinical characteristics of participants at admission

Characteristics	Total (N = 109)	Survival cases (n = 61)	Non-survival (n = 48)	*P-*value
**Age,** mean ± SD, years	65.43 ± 12.84	65.92 ± 13.18	64.81 ± 12.50	0.657
**Sex,** n (%):				
Female	74 (67.9)	40 (65.6)	34 (70.8)	0.706
Male	35 (32.1)	21 (34.4)	14 (29.2)	
**BMI**, mean ± SD, kg/m^2^	23.35 ± 3.29	23.13 ± 3.82	23.50 ± 2.92	0.650
**Current smoker,** n (%):				
Yes	7 (6.4)	2 (3.3)	5 (10.4)	0.131
No	102 (93.6)	59 (96.7)	43 (89.6)	
**Co-morbidities,** n (%):				
Hypertension	46 (42.2)	27 (44.3)	19 (39.6)	0.623
Diabetes	20 (18.3)	14 (23.0)	6 (12.5)	0.162
Coronary heart disease	16 (14.7)	9 (14.8)	7 (14.6)	0.980
Renal insufficiency	8 (7.3)	1 (1.6)	7 (14.6)	0.010
Chronic lung disease	8 (7.3)	5 (8.2)	3 (6.3)	0.699
Cerebrovascular disease	5 (4.6)	1 (1.6)	4 (8.3)	0.097
Malignant tumor	5 (4.6)	2 (3.3)	3 (6.3)	0.462
**Surgery history within 6 months,** n (%)	5 (4.6)	1 (1.6)	4 (8.3)	0.097
**Signs and symptoms,** n (%):				
Any	138 (100)	109 (100)	29 (100)	1.000
Fever	90 (82.6)	46 (75.4)	44 (91.7)	0.026
Highest temperature				0.979
37.3-38.0°C	24 (26.7)	12 (26.1)	12 (27.3)	
38.1-39.0°C	51 (56.7)	26 (56.5)	25 (56.8)	
>39.0°C	15 (16.7)	8 (17.4)	7 (15.9)	
Duration of fever, median (IQR), days	7 (2-10)	5 (1-10)	7 (4-10)	0.040
Cough or sputum production	91 (83.5)	52 (85.2)	39 (81.3)	0.577
Chest distress/dyspnea	67 (61.5)	35 (57.4)	32 (66.7)	0.323
Fatigue	69 (63.3)	37 (60.7)	32 (66.7)	0.518
Breathlessness or wheezing	55 (50.5)	26 (42.6)	29 (60.4)	0.065
Nausea or vomiting	2 (1.8)	2 (3.3)	0 (0.0)	0.205

SD – standard deviation, IQR – interquartile range, BMI – body mass index

The vital signs, laboratory and radiological findings at admission were summarized in Table S1 of the **Online Supplementary Document**. The details of the use of antiviral agents were shown in Table S2 of the **Online Supplementary Document**. The details of other treatments were shown in Table S3 of the **Online Supplementary Document**.

### Effects of antiviral drugs on the in-hospital

As shown in **Table 2**, there were 45 (41.3%) patients treated by oseltamivir, 23 (21.1%) patients treated by arbidol, 16 (14.7%) patients treated by IFN-α, 7 (6.4%) patients treated by LPV/r, 50 (415.9%) patients treated by Ribavirin.

**Table 2:** Effect of antiviral treatments on in-hospital mortality*

Antiviral agents	Total (N = 109)	Survival (n = 61)	Non-survival (n = 48)	Odds ratio (OR)	*P-*value
Abidol	23 (21.1)	6 (9.8)	17 (35.4)	5.027 (1.795-14.074)	0.001
Lopinavir/ritonavir (LPV/r)	7 (6.4)	6 (9.8)	1 (2.1)	0.195 (0.023-1.679)	0.101
Ribavirin	50 (45.9)	30 (49.2)	20 (41.7)	0.738 (0.344-1.582)	0.434
Oseltamivir	45 (41.3)	27 (44.3)	18 (37.5)	0.756 (0.349-1.636)	0.477
Interferon (IFN)-α	16 (14.7)	12 (19.7)	4 (8.3)	0.371 (0.112-1.236)	0.097
Abidol+Oseltamivir	10 (9.2)	2 (3.3)	8 (16.7)	5.900 (1.190-29.242)	0.016
Abidol+IFN	4 (3.7)	3 (4.9)	1 (2.1)	0.411 (0.041-4.085)	0.435
LPV/r+Oseltamivir	4 (3.7)	3 (4.9)	1 (2.1)	0.411 (0.041-4.085)	0.435
LPV/r+IFN	4 (3.7)	3 (4.9)	1 (2.1)	0.411 (0.041-4.085)	0.435

OR – odds ratio, CI – confidence interval

*No patients were treated with Arbidol+LPV/r, Arbidol+Ribavirin, or LPV/r+Ribavirin, or Ribavirin+IFN.

We found no significant association between LPV/r treatment and the in-hospital mortality (OR = 0.195, 95% CI = 0.023-1.679). Additionally, ribavirin (OR = 0.738, 95% CI = 0.344-1.582), oseltamivir (OR = 0.765, 95% CI = 0.349-1.636), and IFN-α (OR = 0.371, 95% CI = 0.112-1.236) were not associated with the in-hospital mortality. However, the use of arbidol was associated with an increased the in-hospital mortality (OR = 5.027, 95% CI = 1.795-14.074). In addition, the combined therapy of arbidol and oseltamivir was also associated with an increased mortality (OR = 5.900, 95% CI = 1.190-29.247). Nevertheless, the combined therapy of arbidol-IFN (OR = 0.411, 95% CI = 0.041-4.085) was not associated with survival in hospital, which was similar with LPV/r-oseltamivir (OR = 0.411, 95% CI = 0.041-4.085) and LPV/r-IFN (OR = 0.411, 95% CI = 0.041-4.085).

**Figure 1:**
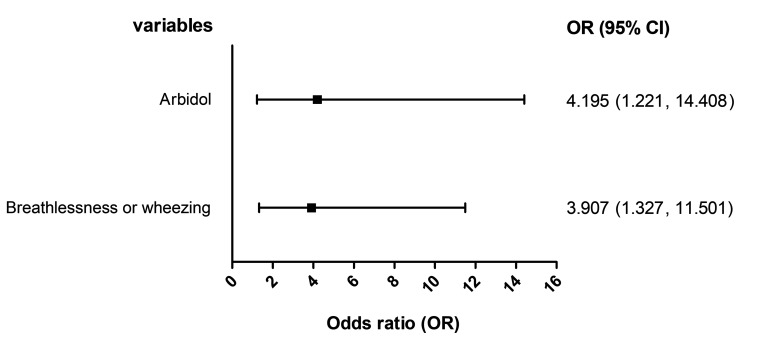
Multiple logistic regression analysis on in-hospital mortality.

A multiple logistic regression analysis was performed to adjust for the confounding of clinical characteristics and other treatments that were founded to be associated with outcome in the above-mentioned results. As listed in **Figure 1**, the multiple logistic regression also identified a significant association of the in-hospital mortality with arbidol (adjusted OR = 4.195, 95% CI = 1.221-14.408), as well as breathlessness or wheezing at admission (adjusted OR = 3.907, 95% CI = 1.327-11.501). While no significant association was observed regarding other antiviral drugs.

### Effects of antiviral drugs on the ICU mortality

**Figure 2:**
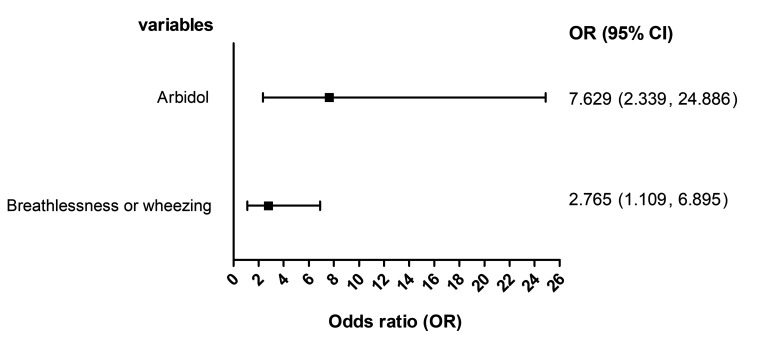
Multiple logistic regression analysis on intensive care unit (ICU) mortality.

As shown in **Table 3**, the use of LPV/r (OR = 0.556, 95% CI = 0.466-0.663) and IFN-α (OR = 0.670, 95% CI = 0.495-0.906) were associated with a decreased ICU mortality. However, arbidol treatment was associated with an increased ICU mortality (OR = 4.490, 95% CI = 1.655-12.180). However, our results suggested that the ICU mortality was not associated with ribavirin (OR = 0.672, 95% CI = 0.306-1.473), and oseltamivir (OR = 0.765, 95% CI = 0.348-1.679). In addition, the administration of arbidol-oseltamivir was not associated with an increased ICU mortality (OR = 3.721, 95% CI = 0.905-15.295). The combined use of arbidol-IFN-α LPV/r-oseltamivir, or LPV/r-IFN-α was not associated with the ICU mortality, for which the OR was not available due to the small number of patients receiving combined antiviral treatment. The multiple logistic regression found a significant association of the ICU mortality with the use of arbidol (adjusted OR = 7.629, 95% CI = 2.339-24.886) and breathlessness or wheezing at admission (adjusted OR = 2.765, 95% CI = 1.109-6.895) (**Figure 2**).

**Table 3:** Effect of antiviral treatments on ICU mortality*

Antiviral agents	Total (N = 106)	Survival (n = 62)	Non-survival (n = 44)	Odds ratio (OR)	*P-*value
Abidol	23 (21.7)	7 (11.3)	16 (36.4)	4.490 (1.655-12.180)	0.002
Lopinavir/ritonavir (LPV/r)	7 (6.6)	7 (11.3)	0 (0.0)	0.556 (0.466-0.663)	0.021
Ribavirin	47 (44.3)	30 (48.4)	17 (38.6)	0.672 (0.306-1.473)	0.319
Oseltamivir	45 (42.5)	28 (45.2)	17 (38.6)	0.765 (0.348-1.679)	0.503
Interferon (IFN)-α	16 (15.1)	13 (21.0)	3 (6.8)	0.670 (0.495-0.906)	0.045
Abidol+Oseltamivir	10 (9.4)	3 (4.8)	7 (15.9)	3.721 (0.905-15.295)	0.055
Abidol+IFN	4 (3.8)	4 (6.5)	0 (0.0)	-	0.230
LPV/r+Oseltamivir	4 (3.8)	4 (6.5)	0 (0.0)	-	0.230
LPV/r+IFN	4 (3.8)	4 (6.5)	0 (0.0)	-	0.230

ICU – intensive care unit, OR – odds ratio, CI – confidence interval

*No patients were treated with Arbidol+LPV/r, Arbidol+Ribavirin, or LPV/r+Ribavirin, or Ribavirin+IFN.

### Onsets of complications

In this study, we documented the onsets of 11 complications (ie, respiratory failure, ARDS, sepsis, acute cardiac injury, acute liver injury, acute kidney injury, septic shock, DIC, arrhythmia, gastrointestinal bleeding, ACVD) among the patient of severe COVID-19. The frequency of onset of ARDS in the survival group (39, 63.9%) was significantly higher than that in the non-survival group (20, 41.7%), meanwhile the frequency of ACVD among survival patients (5, 8.2%) was also higher than that of fatal ones (0, 0%). No significant difference was observed in other complications (**Table 4**).

**Table 4:** Onsets of complications during hospitalization (n, %)

Complications	Total (N = 109)	Survival (n = 61)	Non-survival (n = 48)	*P-*value
Any type	100 (91.7)	58 (95.1)	42 (87.5)	0.153
Respiratory failure	85 (78.0)	49 (80.3)	36 (75.0)	0.505
ARDS	59 (54.1)	39 (63.9)	20 (41.7)	0.021
Sepsis	44 (40.4)	26 (42.6)	18 (37.5)	0.588
Acute cardiac injury	30 (27.5)	19 (31.1)	11 (22.9)	0.340
Acute liver injury	29 (26.6)	20 (32.8)	9 (18.8)	0.100
Acute kidney injury	26 (23.9)	18 (29.5)	8 (16.7)	0.118
Septic shock	22 (20.2)	16 (26.2)	6 (12.5)	0.076
Arrhythmia	10 (9.2)	7 (11.5)	3 (6.3)	0.348
DIC	6 (5.5)	4 (6.6)	2 (4.2)	0.587
Gastrointestinal bleeding	6 (5.5)	5 (8.2)	1 (2.1)	0.165
ACVD	5 (4.6)	5 (8.2)	0 (0)	0.042

ARDS – acute respiratory distress syndrome, DIC – disseminated intravascular coagulation, ACVD – acute cerebrovascular disease

The onsets of complications in patients received different antiviral drugs were analyzed. As shown in Table S4 of the **Online Supplementary Document**, oseltamivir was associated with high incidence of respiratory failure, ARDS and acute kidney injury, and low incidence of gastrointestinal bleeding, ACVD, acute cardiac injury, and acute liver injury. Arbidol was associated with a decrease in the development of sepsis, whereas Ribavirin was associated with a higher incidence of sepsis. This finding was also subject to small numbers of patients who received LPV/r or IFN-α.

## DISCUSSION

This retrospective study observed a survival rate of 56.0% among severe COVID-19 patients who received different antiviral treatments during hospitalization. The treatment of arbidol resulted in increased ICU and in-hospital mortality. Ribavirin, oseltamivir and interferon showed no beneficial effects for severe COVID-19 patients. LPV/r was likely to improve the survival rate while the difference was not statistically significant.

As the situation of COVID-19 epidemic is still remaining severe worldwide, identification of specific effective treatment is urgently necessary. In addition to the combined therapies of symptomatic treatment, prevention of complications, treatment of accompanying diseases, prevention of secondary infections, and organ function support, antiviral treatment has been considered as a crucial approach for patients’ recovery [16]. There have been more than 300 clinical trials targeting the potential of COVID-19 therapy ongoing, some of them will be released the findings in the next couple of months [17]. In the first hit city by COVID-19, we have used several antiviral agents (ie, ribavirin, LPV/r, oseltamivir, arbidol and IFN) for the management of patients in our hospitals in Wuhan by reference with the official guideline, as well as the prescription used to treat severe acute respiratory syndrome (SARS), Middle East Respiratory Syndrome (MERS), influenza and human immunodeficiency virus (HIV) [18,19].

The compound of lopinavir/ritonavir (LPV/r) is commonly used to treat HIV infection. LPV/r has been approved to be successful on curing SARS and MERS through in vitro, animal and clinical studies [20]. LPV/r (200 mg/50 mg per capsule, 2 capsules each time, twice per day for adults) was recommended officially in the early released version of NHC guidelines and the updated versions. However, a randomized trial enrolled 199 severe COVID-19 patients did not observe significant benefit in LPV/r treated group as compared to standard care. In addition, another trial within 44 patients with mild/moderate COVID-19 showed no benefit in the time to viral clearance or clinical progression to severe disease within LPV/r treated group [21]. In this retrospective study, there was no statistically significant association between LPV/r treatment and improved outcome, which was in line with these previous trails.

Arbidol is a small indole-derivative molecule that has been licensed for prophylaxis and treatment of influenza virus and other respiratory viral infections in Russia and China. A case series study reported that four patients with mild or severe COVID-19 recovered after receiving treatment of arbidol combined with a traditional Chinese medicine [22]. Zhu and colleagues found that arbidol monotherapy was superior to lopinavir/ritonavir in treating COVID-19 patients [23]. In addition, it has been demonstrated that the combination of arbidol and LPV/r is superior to LPV/r alone [24]. Based on these evidences, arbidol (200 mg for adults, three times per day) has been recommended in the sixth version of NHC guideline on February 18, 2020 [10]. In the present study, we found that arbidol induced an increase in-hospital mortality among patients with severe COVID-19 compared individuals who did not receive arbidol. In addition, the combination of arbidol with oseltamivir also led to an increased mortality.

Ribavirin is a synthetic nucleoside antiviral agent with a broad-spectrum antiviral activity against both DNA and RNA viruses, which is phosphorylated in virus infected cells, and its product acts as a competitive inhibitor of virus synthetase. Ribavirin interferes with early viral transcription and hinds the synthesis of ribonucleoproteins, playing a role in restrain of virus replication [25]. Combination of ribavirin with other antiviral agents has been also recommended by the NHC of China [10]. However, we found no beneficial effects of ribavirin on the prognosis of severe COVID-19 in this study. Oseltamivir is one of well-known neuraminidase inhibitors that was developed for control influenza virus [26]. A study on 393 COVID-19 patients treated with oseltamivir found that oseltamivir administration showed no efficacy in the improvement of ICU admission rate, the need for ventilator, and death rate [9]. Our findings indicated that oseltamivir was not associated with improved outcome. Interferons (IFNs) was also recommended for the treatment of COVID-19 in China. However, there are no academic evidences concerning its effectiveness in treating COVID-19 patients. Our findings demonstrated that neither IFN-α therapy nor combination with arbidol was not associated with improvement of survival.

Severe COVID-19 patients suffer from high incidences complications such as respiratory failure, ARDS, sepsis, acute cardiac injury, acute liver injury, acute kidney injury, septic shock, DIC, arrhythmia, gastrointestinal bleeding, and ACVD [27]. Our study indicated that individuals discharged alive suffered high frequencies of ARDS and ACVD during hospitalization than fatal cases. With regard to the onsets of complications in patients received different antiviral drugs, patients treated with oseltamivir suffered more complications (eg, respiratory failure, ARDS and acute kidney injury). Arbidol was associated with a decreased incidence of sepsis, whereas Ribavirin was associated with a higher incidence of sepsis.

### Limitations

Our study has several limitations that are commonly involved in retrospective studies. First, the assignment of the antiviral and supportive treatments was subject to the situation and environment of the hospital, which might influence clinical decision-making; Second, the patients were not allocated randomly, and the demographical and clinical characteristics at baseline were not entirely balanced between groups; Third, the use of other pharmacologic interventions were arranged on the basis on the condition of specific patients, which might bias the assessment of the efficacies of antiviral medications; Fourthly, although the multiple logistic regression analysis was conducted to adjust underlying confounders, the potential bias and interactions of different medications were not able to eliminated completely; Finally, and probably the most important limitation is that the effects of antiviral drugs on clinical course of severe COVID-19 cases had not been investigated in the present study. Although we found no beneficial effects of antiviral drugs in the in-hospital mortality, whether the use of antiviral drugs shorten the clinical course remains unclear.

## CONCLUSIONS

Our results suggested that LPV/r, oseltamivir, IFN-α, ribavirin, or oseltamivir treatment shows no beneficial effects for severe COVID-19 patients, whereas the use of arbidol is associated with an increased in-hospital mortality. Therefore, the current and future situations make it a serious concern to pursue more actions to identify specific antiviral agent against COVID-19.

## Additional material

Online Supplementary Document
