# Supplementary material for: Arbidol is associated with increased in-hospital mortality among 109 patients with severe COVID-19: A multicenter, retrospective study
Source: J Glob Health. 2021 Jul 17;11:05017. doi: 10.7189/jogh.11.05017 (PMC8284661; doi:10.7189/jogh.11.05017)
Supplement: Online Supplementary Document [file jogh-11-05017-s001.pdf]

[Supplementary tables]

Table S1 Vital signs, laboratory and radiological findings at admission

Variables	Total (N=109)	Survival (n=61)	Non-survival (n=48)	*P-value*
**Vital signs**				
Systolic blood pressure (mmHg, IQR)	130 (120-140)	131 (120-140)	130 (120-140)	
>140	1 (0.9）	0 (0.0)	1 (2.1)	0.470
<90	23 (21.1)	14 (23.0)	9 (18.8)	
Diastolic blood pressure (mmHg, IQR)	80 (72-85)	80 (75-87.75)	78 (70-82)	
>90	6 (5.5)	2 (3.3)	4 (8.3)	0.433
<60	9 (8.3)	6 (9.8)	3 (6.3)	
Heart rate (beats per minute, IQR)	90 (80-101)	88 (80-99)	98 (80-102)	
>100	30 (27.5)	13 (21.3)	17 (35.4)	0.102
Axillary temperature (°C, IQR)	36.8 (36.6-37.7)	36.8 (36.50-37.58)	36.9 (36.6-37.8)	
>37.3	36 (33.0)	19(31.1)	17 (35.4)	0.638
Respiratory rate (breaths per minute, IQR)	23 (20-26)	21.5 (20-26)	25 (20-28)	
21-29	49 (45.4)	23 (37.7)	26 (55.3)	0.075
≥ 30	16 (14.8)	8 (13.1)	8 (17.0)	
Oxygen saturation (%, IQR)	89 (85-94)	91 (85-96.75)	88 (84-90)	
≤93%	80 (73.4)	38 (62.3)	42 (87.5)	0.003
**Laboratory findings**				
Leucocytes (10^9/L, IQR)	6.71 (4.74-10.9)	6.17 (4.60-9.69)	7.8 (5.2-11.5)	
>9.5	33 (30.6)	15 (25.0)	18 (37.5)	0.195
<3.5	9 (8.3)	7(11.7)	2(4.2)	
Neutrophils (10^9/L, IQR)	6.09 (3.44-9.93)	5.82 (3.19-8.76)	6.40 (3.58-10.11)	
>6.3	52 (48.1)	26 (43.3)	26 (54.2)	0.509
Lymphocytes (10^9/L, IQR)	0.58 (0.36-0.89)	0.54 (0.36-0.81)	0.72 (0.43-0.96)	
< 1.0	90 (83.3)	51 (85)	39 (81.3)	0.603
≥ 1.0	18 (16.7)	9 (15)	9 (18.7)	
Monocyte (10^9/L, IQR)	0.31 (0.15-0.47)	0.27 (0.15-0.42)	0.33 (0.14-0.51)	
>0.6	15 (13.9)	6 (10)	9 (18.8)	0.376
<0.1	19 (17.6)	12 (20)	7 (14.6)	
Erythrocyte (10^{12}/L, IQR)	4.05 (3.66-4.51)	4.07 (3.68-4.36)	4 (3.46-4.62)	
<4.3	71 (65.7)	42 (70)	29 (60.4)	0.468
Hemoglobin (g/L, IQR)	121 (108.25-133.50)	122 (109-134)	120 (108-133.50)	
<130	77 (71.3)	43 (71.7)	34 (70.8)	0.650
Platelet count (10^9/L, IQR)	173 (127-240.25)	184 (142-256)	158 (110-211.50)	
<125	26 (24.1)	12 (20)	14 (29.2)	0.333
Procalcitonin (ng/mL, IQR)	0.28 (0.06-1.29)	0.64 (0.06-1.32)	0.16 (0.06-1.26)	
>0.5	46 (44.2)	30 (50.8)	16 (35.6)	0.120
IL-6 (pg/mL, IQR)	0 (0-94.07)	0 (0-94.07)	0 (0-4.14)	
>7.0	3 (9.4)	0 (0)	3 (20)	0.053
ESR (mm/h, IQR)	43 (23-54.50)	40 (22-61)	46 (22-55)	
>15.0	16 (88.9)	4 (80.0)	12 (92.3)	0.457
APTT (s, IQR)	30 (28.3-37)	32.75 (28.75-37.68)	30 (25.3-30.7)	
>43.5	1 (5.9)	1 (7.1)	0 (0.0)	0.831
PT (s, IQR)	13.8 (12.05-16.65)	13.2 (11.90-16.88)	14 (13.1-15.4)	
>16.0	5 (29.4)	5 (35.7)	0 (0)	0.218
D-dimer (ng/mL, IQR)	1.11 (0.41-5.03)	0.92 (0.37-2.94)	1.04 (0.45-5.61)	
>500.0	2 (2.2)	0 (0.0)	2 (4.3)	0.148
Fibrinogen (mg/dl, IQR)	3.85 (2.86-5.03)	3.85 (2.90-4.93)	3.86 (2.66-5.07)	
>498	3 (3)	1 (1.9)	2 (4.3)	0.897
<238	92 (92.9)	50 (94.3)	42 (91.3)	
ALT (U/L, IQR)	35 (23-52)	36 (23-47.5)	33.95 (20.8-56.18)	
>50.0	28 (26.2)	13 (22)	15 (31.3)	0.320

AST (U/L, IQR)	41 (28.6-59.9)	37 (27-53)	44.5 (31.9-70)	
>40.0	54 (50.5)	26 (44.1)	28 (58.3)	0.320
TBLI (μmol/L, IQR)	10.5 (7.5-16.51)	11.16 (7.49-15.10)	10.35 (5.40-19.70)	
>21.0	15 (14)	4 (6.8)	11 (22.9)	0.002
Albumin (g/L, IQR)	33.1 (29.8-36.2)	34.5 (32.93-38.85)	32.80 (28.35-36.15)	
<40.0	97 (90.7)	51 (86.4)	46 (95.8)	0.097
Creatinine (μmol/L, IQR)	78.8 (58.8-105.4)	78.65 (63.43-91.30)	79.55 (60.70-146.93)	
>104.0	29 (27.1)	12 (20.3)	17 (35.4)	0.150
BUN (mmol/L, IQR)	6.6 (4.9-8.9)	5.25 (4.46-6.48)	7.28 (5.32-17.33)	
>7.6	37 (34.6)	11 (18.6)	26 (54.2)	0.001
hs-cTnI (pg/mL, IQR)	0.02 (0.01-0.10)	0.02 (0.01-0.06)	0.04 (0.01-0.18)	
>26.2	4 (4.7)	1 (2.3)	3 (7)	0.306
Blood glucose (mmol/L, IQR)	6.9 (5.89-10.40)	6.66 (5.71-9.75)	7.78 (6.41-10.64)	
>6.1	66 (69.5)	37 (62.7)	29 (80.6)	0.036
pH (IQR)	7.42 (7.36-7.46)	7.42 (7.39-7.45)	7.44 (7.30-7.48)	
>7.45	26 (28.6)	13 (24.5)	13 (34.2)	0.038
<7.35	20 (22)	8 (15.1)	12 (31.6)	
PaO_2 (mmHg, IQR)	68.4 (47-81.6)	73 (60.75-89.65)	59.05 (34-75)	
<80	65 (71.4)	33 (62.3)	32 (84.2)	0.055
$PaCO_2$ (mmHg, IQR)	39.10 (33.70-47.70)	36.45 (31.98-40.45)	46 (34.75-63.50)	
>45	28 (31.1)	11 (20.8)	17 (45.9)	0.016
HCO_3^- (mmol/L, IQR)	24.75 (20.90-20.18)	25.50 (23.35-28.98)	27.70 (18.10-31.15)	
>27.3	38 (42.2)	21 (39.6)	17 (45.9)	0.001
<21.4	22 (24.4)	7 (13.2)	15 (40.5)	
Lactate (mmol/L, IQR)	2.26 (1.50-3.15)	1.88 (1.40-2.48)	3.10 (2.19-3.85)	
>1.6	62 (69.7)	35 (61.4)	27 (84.4)	0.066

IQR, interquartile range

Table S2 Antiviral treatments of COVID-19 cases

**Antiviral agents**	**Survival**	**Non-survival**	***P-value***
**Abidor**	6 (9.8)	17 (35.4)	0.001
Dose: median (min−max), g/day	0.2 (0.2-0.2)	0.2 (0.2-0.2)	-
**Lopinavir/ritonavir (LPV/r)**	6 (9.8)	1 (2.1)	0.101
Dose: median (min−max), mg/day	400 (200-400)	400 (200-400)	-
**Oseltamivir** (n, %)	27 (44.3)	18 (37.5)	0.477
Dose: median (min−max), mg/day	150 (150-150)	150 (150-150)	-
**Ribavirin**	30 (49.2)	20 (41.7)	0.434
Dose: median (Min~Max), g/day	0.9 (0.8-1.0)	0.9 (0.8-1.0)	
**Interferon (IFN)-α**	12 (19.7)	4 (8.3)	0.097
Dose: median (min−max), mIU/day	4 (3-5)	4 (3-5)	-

mIU, million international units

Table S3 Other treatments for severe COVID-19 cases

**Treatment**	**Survival (n=61)**	**Non-survival (n=48)**	***P-value***
**Antibiotics**	58 (95.1)	48 (100)	0.119
**Glucocorticoids**	37 (60.7)	43 (89.6)	0.001
Initial dose, median (IQR), mg/qd	40 (40-80)	40 (40-80)	0.341
Duration of therapy, median (IQR), d	3 (2-7)	5 (2-7)	0.131
**Vasoactive drugs**	12 (19.7)	20 (41.7)	0.012
Duration of therapy, median (IQR), d	5.00 (3.25-9.25)	2.50 (0.00-7.75)	0.125
**High-flow oxygen therapy**	17 (27.9)	14 (29.2)	0.881
Duration of therapy, median (IQR), d	4.00 (1.00-6.75)	4.00 (1.00-6.75)	0.882
**Non-invasive ventilation**	7 (11.7)	24 (50)	<0.001
Duration of therapy, median (IQR), d	8.5 (0.25-14.00)	3.50 (0.00-12.75)	0.627
**Invasive mechanical ventilation**	18 (29.5)	38 (79.2)	<0.001
Duration of therapy, median (IQR), d	8.50 (5.00-15.00)	8.00 (0.00-14.25)	0.549
**Prone position ventilation**	5 (8.2)	4 (8.3)	0.979
**CRRT**	0 (0)	4 (8.3)	
Duration of therapy, median (IQR), d		3.00 (1.25-7.75)	0.022
**ICU admission**	60 (98.4)	46 (95.8)	0.432
Length of stay, median (IQR), d	9.50 (3.50-13.75)	7.00 (3.00-14.25)	0.459

IQR, interquartile range; CRRT, continuous renal replacement therapy; ICU, intensive care unit;

Table S4 Onsets of complications for different antiviral treatment (n, %)

Complications	Arbidol (n=23)	Lopinavir/ritonavir (n=7)	Ribavirin (n=50)	Oseltamivir (n=45)	Interferon-α (n=16)
Respiratory failure	19 (82.6)	3 (42.9)*	36 (72.0)	41 (91.1)*	10 (62.5)
ARDS	10 (43.5)	2 (28.6)	20 (40.0)*	35 (77.8)*	10 (62.5)
Sepsis	2 (8.7)*	1 (14.3)	35 (70.0)*	8 (17.8)*	4 (25.0)
Acute cardiac injury	2 (8.7)	2 (28.6)	12 (24.0)*	8 (17.8)*	4 (25.0)
Acute liver injury	6 (26.1)	2 (28.6)	6 (12.0)	19 (42.2)	7 (43.8)
Acute kidney injury	3 (13.0)	0 (0.0)	13 (26.0)	15 (33.3)*	4 (25.0)
Septic shock	3 (13.0)	0 (0.0)	9 (18.0)	15 (33.3)	4 (25.0)
DIC	3 (13.0)	0 (0.0)	2 (4.0)	4 (8.9)	1 (6.2)
Gastrointestinal bleeding	1 (4.3)	0 (0.0)	1 (2.0)	5 (11.1)*	1 (6.2)
ACVD	0 (0.0)	0 (0.0)	0 (0.0)*	5 (11.1)*	0 (0.0)
Arrhythmia	2 (8.7)	2 (28.6)	0 (0.0)*	7 (15.6)	2 (12.5)

ARDS, Acute respiratory distress syndrome; DIC, disseminated intravascular coagulation; ACVD, Acute cerebrovascular disease;

* Statistically significant compared to patients who were not treated the antiviral agent.
